# Does Modality of Survey Administration Impact Data Quality: Audio Computer Assisted Self Interview (ACASI) Versus Self-Administered Pen and Paper?

**DOI:** 10.1371/journal.pone.0008728

**Published:** 2010-01-15

**Authors:** William M. Reichmann, Elena Losina, George R. Seage, Christian Arbelaez, Steven A. Safren, Jeffrey N. Katz, Adam Hetland, Rochelle P. Walensky

**Affiliations:** 1 Department of Orthopedic Surgery, Brigham and Women's Hospital, Boston, Massachusetts, United States of America; 2 Department of Biostatistics, Boston University School of Public Health, Boston, Massachusetts, United States of America; 3 Center for AIDS Research, Harvard Medical School, Boston, Massachusetts, United States of America; 4 Department of Epidemiology, Harvard School of Public Health, Boston, Massachusetts, United States of America; 5 Department of Emergency Medicine, Brigham and Women's Hospital, Boston, Massachusetts, United States of America; 6 Department of Psychiatry, Massachusetts General Hospital, Boston, Massachusetts, United States of America; 7 Division of Rheumatology, Immunology and Allergy, Brigham and Women's Hospital, Boston, Massachusetts, United States of America; 8 Division of Infectious Diseases, Brigham and Women's Hospital, Boston, Massachusetts, United States of America; 9 Division of Infectious Diseases, Massachusetts General Hospital, Boston, Massachusetts, United States of America; 10 Division of General Medicine, Massachusetts General Hospital, Boston, Massachusetts, United States of America; University of Toronto, Canada

## Abstract

**Background:**

In the context of a randomized controlled trial (RCT) on HIV testing in the emergency department (ED) setting, we evaluated preferences for survey modality and data quality arising from each modality.

**Methods:**

Enrolled participants were offered the choice of answering a survey via audio computer assisted self-interview (ACASI) or pen and paper self-administered questionnaire (SAQ). We evaluated factors influencing choice of survey modality. We defined unusable data for a particular survey domain as answering fewer than 75% of the questions in the domain. We then compared ACASI and SAQ with respect to unusable data for domains that address sensitive topics.

**Results:**

Of 758 enrolled ED patients, 218 (29%) chose ACASI, 343 chose SAQ (45%) and 197 (26%) opted not to complete either. Results of the log-binomial regression indicated that older (RR = 1.08 per decade) and less educated participants (RR = 1.25) were more likely to choose SAQ over ACASI. ACASI yielded substantially less unusable data than SAQ.

**Conclusions:**

In the ED setting there may be a tradeoff between increased participation with SAQ versus better data quality with ACASI. Future studies of novel approaches to maximize the use of ACASI in the ED setting are needed.

## Introduction

Audio computer assisted self-interview (ACASI) is a survey modality that is frequently used in healthcare and clinical research settings to collect patient information. It is most useful when sensitive information is requested and subjects perceive that they may be judged by their responses [1]–[13]. Randomized studies of ACASI have observed that people are more likely to report “risky” behaviors, such as unprotected sex and needle sharing, in ACASI based surveys than in face-to-face personal interviews [1], [2], [6], [7], [12]. When ACASI has been compared with self-administered questionnaires (SAQ), the results have been mixed [5], [9], [10]. Some studies document no increased reporting of risky behaviors [5], while others have found an increased reporting of risky behaviors with ACASI [9]. Others have found that gender may modify the effect of survey modality on the amount of reporting of risky behaviors [10]. None of these studies have compared ACASI and SAQ in the emergency department (ED) setting.

Within the context of a randomized controlled trial (RCT) of HIV screening programs in the ED, we requested from subjects sensitive information via ACASI. However, in the first two months of our trial many participants requested to complete our survey via the more traditional pen and paper self-administered questionnaire (SAQ). Given this experience and the paucity of literature examining subject preferences and survey completeness when both SAQ and ACASI are offered, our objective was to more formally analyze differences in preferences and survey completion. Accordingly, we first sought to identify socio-demographic factors that might affect subjects' preference for SAQ vs. ACASI. Second, we sought to evaluate the impact of survey modality on the completeness of the data across different domains that address sensitive information.

## Methods

### Study Overview

The Universal Screening for HIV in the Emergency Room (USHER) trial was a randomized controlled trial designed to evaluate alternative approaches to routine HIV testing in the ED (ClinicalTrials.Gov: #NCT00502944) [14]. Within the context of this study, enrolled participants were asked to complete an 85-item survey that assessed their attitudes toward HIV testing and their knowledge about HIV infection [15], and inquired about their history of HIV testing, sexual behavior, mental health [16]–[18], alcohol [19], and drug use. Where possible the survey used standardized, validated items and scales [15]–[19]. Participants who were enrolled from April 1, 2007 to August 16, 2007 were offered the choice of completing the research survey via the ACASI or SAQ. The consent form for the study was uniformly standardized and did not contain information on survey modality.

The study randomized participants to a rapid oral HIV testing to be offered and conducted by a member of their health care team (provider arm) or by an independent HIV counselor (counselor arm) [20]. Randomization occurred after the participant agreed to enrollment. Upon randomization, but generally prior to HIV test offer, the participant was asked his/her preference for survey modality; the survey was provided according to patients stated preference. Minimizing disruptions in patient ED flow was critical for study success; on occasion, therefore, participants may have been interrupted during the survey so that the HIV screening test – which takes 20 minutes to develop – could be offered and conducted. The Brigham and Women's Hospital institutional review board approved the study, and the study was overseen by a data safety monitoring board.

### Study Setting and Population

The Brigham and Women's Hospital ED is a major level 1 trauma center with over 56,000 patient visits per year. To be eligible for participation in the USHER trial, patients had to be: 1) from 18 to 75 years old, 2) fluent in English or Spanish, 3) assigned an emergency severity index (ESI) score of 3, 4, or 5 (range: 1–5; 1 = most severe, 5 = least severe) [21]–[23], 4) not known to be HIV-infected, and 5) not be in pre-natal care. Enrolled participants provided written informed consent and were not paid for participation.

### Survey Development

Survey questions were developed at a 4^th^ grade standard reading level and were reviewed by a literacy expert for clarity and comprehensibility. Both survey modalities asked identical questions presented in the same order.

The ACASI program was developed using the Questionnaire Development System (QDS™) software (NOVA Research Company). During the enrollment process, participants were told that a laptop would be wheeled into their room if they were to choose the computer version of the survey. Participants were instructed on how to use the laptop and that no prior computer experience was necessary for its completion. Research assistants were available for assistance in completing the survey at any time. The ACASI survey used in the USHER trial was available to participants in both English and Spanish, each in a male or female voice (four different combinations); participants completing the survey via ACASI had their choice among the four versions. A single question appeared on the screen at a time, and the responses were highlighted as they were read. Participants who chose ACASI completed the survey on a portable laptop on a bedside table in their ED room. Participants had the option to pause and resume the ACASI program as needed during their ED stay. They could also skip questions or go back and change their answer to previously answered questions. Automated skips were built into the program where appropriate to shorten the length of time needed to complete the survey. For example, if the participants answered to ‘No’ to being a current smoker, the ACASI program would skip the question about the number of cigarettes smoked per day.

The SAQ version of the survey was also available to participants in both English and Spanish. The 18-page survey allowed participants to mark their response by checking a box. Participants could pause and skip questions as needed. The automated skips that were built into the ACASI program were written on the page. For example, if the participant responded ‘No’ to the smoking question, he/she would see a note that instructed to advancement to the next applicable question.

### Data Elements

Demographic data, including age, gender, race/ethnicity, primary language, and education were collected from the participants by the research assistant at the time they were offered enrollment, which was prior to being offered the survey. The survey collected data on seven domains, which included, attitudes toward HIV testing; knowledge about HIV [15]; socioeconomic status and medical history; history of HIV testing; sexual behavior, including the number of different sexual partners in the past 12 months and the past 3 months; mental health [16]–[18]; and smoking status, alcohol usage [19], and illicit drug usage. Data on HIV knowledge was collected using the validated 18-item HIV Knowledge Questionnaire, which has been found to be internally consistent and suitable for low-literacy populations [15]. Mental health was assessed using the validated Center for Epidemiological Studies-Depression (CES-D) scale, used to screen for depressive symptoms. The CES-D scale is a validated survey that has shown good measurement properties (Cronbach's alpha = 0.9) [16]–[18]. Alcohol usage was evaluated with the validated Alcohol Use Disorders Test (AUDIT), developed by the World Health Organization [19]. Further details regarding the data elements can be found in Appendix S1.

### Defining Unusable Data in Survey Response

We defined the data for a specific domain, such as sexual behavior or depression, as ‘unusable’ if the respondent completed less than 75% of the questions in that particular domain. We chose a 75% threshold because it was the minimum to allow a validated score to be constructed based on the data provided by a particular respondent. We used 90% as an alternative threshold in a sensitivity analysis.

### Statistical Analysis

All analyses were conducted using SAS version 9.1 (Cary, NC). Means and standard deviations were calculated to describe continuous variables; proportions were calculated for categorical variables. Log-binomial regression was performed to calculate both unadjusted and adjusted relative risks (RR) of choosing the SAQ version of the survey over the ACASI. We chose log-binomial regression because it provides better estimates of the RR than does standard logistic regression when the prevalence of the outcome is greater than 10%. Ninety-five percent confidence intervals were constructed for both unadjusted and adjusted RRs.

The analysis examining the impact of survey modality on the magnitude of unusable data within across four specific domains (sexual history; depression; alcohol use; and illicit drug use) contained several steps. First, every subject was assigned a propensity score, defined as the probability of choosing the SAQ version of the survey given a set of covariates, including age, gender, race, language, education, religion, income, employment status, comorbidity, and health care utilization. We used the propensity score to adjust for multiple confounders [24]. The next step included building a log-binomial regression model to evaluate the impact of survey modality on the amount of unusable data (less than 75% complete for a specific domain), after adjusting for the propensity score. We divided the propensity score into quintiles and adjusted for the quintile of the propensity score in the log-binomial regression model that evaluated the impact of survey modality on the amount of unusable data. As a sensitivity analysis, we repeated the above analyses using 90% as the threshold for unusable data.

## Results

### Sample

From April 1 to August 16, 2007, 758 patients were enrolled in USHER Trial when both the ACASI and SAQ were offered. Of those enrolled, 561 (74%) patients agreed to answer the survey; 218 (39%) chose the ACASI, while 343 (61%) chose the SAQ.

The mean age of all participants enrolled was 37 years (standard deviation of 13). Two hundred ninety-three (39%) were non-Hispanic white, 147 (20%) were non-Hispanic black, 231 (31%) were Hispanic, and 72 (10%) were classified as other. A majority of the participants were female (64%) and reported English as their primary language (73%). Two hundred ninety (38%) participants had a high school degree or less, while 262 (35%) had at least a college degree. The demographic features were examined by survey completion mode in Table 1.

**Table 1:** Demographic Data by Patient Preference for Survey Modality.

	ACASI (n = 218, 29%)	SAQ (n = 343, 45%)	Not Done (n = 197, 26%)	Entire Sample (n = 758)
Mean Age (SD)	34.8 (12.1)	37.8 (13.7)	38.0 (14.2)	37.0 (13.4)
Race				
Non-Hispanic White	108 (49.5%)	114 (34.3%)	71 (36.8%)	293 (39.4%)
Non-Hispanic Black	36 (16.5%)	73 (22.0%)	38 (19.7%)	147 (19.8%)
Hispanic	54 (24.8%)	112 (33.7%)	65 (33.7%)	231 (31.1%)
Other	20 (9.2%)	33 (9.9%)	19 (9.8%)	72 (9.7%)
Gender				
Male	87 (39.9%)	118 (34.4%)	68 (34.5%)	273 (36.0%)
Female	131 (60.1%)	225 (65.6%)	129 (65.5%)	485 (64.0%)
Language				
English	172 (79.3%)	247 (72.4%)	134 (68.0%)	553 (73.2%)
Spanish	33 (15.2%)	77 (22.6%)	49 (24.9%)	159 (21.1%)
Other	12 (5.5%)	17 (5.0%)	14 (7.1%)	43 (5.7%)
Education				
Less than HS	18 (8.3%)	53 (15.5%)	45 (22.8%)	116 (15.3%)
HS Degree/GED	35 (16.2%)	94 (27.4%)	45 (22.8%)	174 (23.0%)
Some College	68 (31.5%)	90 (26.2%)	46 (23.4%)	204 (27.0%)
College Degree	62 (28.7%)	67 (19.5%)	36 (18.3%)	165 (21.8%)
Some Post-College/Post-College Degree	33 (15.3%)	39 (11.4%)	25 (12.7%)	97 (12.8%)

Where SD stands for standard deviation, HS for high school, GED for General Equivalency Diploma, ACASI for audio computer-assisted self-interview, and SAQ for self-administered questionnaire.

### Associations with Survey Modality

Demographic variables (age, race, and education) were associated with survey modality, while other self-reported psychosocial variables were not (Table 2). Results of the multivariable log-binomial regression confirmed the findings of the unadjusted associations (Table 2). The multivariable log-binomial regression showed that participants who were older (RR = 1.08; 95% CI: 1.03, 1.13; per 10-year increase in age), non-white (1.26; 95% CI: 1.08, 1.47), and having a highest education attainment of high school degree or less (RR = 1.25; 95% CI: 1.09, 1.43) were more likely to choose the paper SAQ over the ACASI version of the survey (Table 2).

**Table 2:** Unadjusted and adjusted associations examining the relationship between patient survey modality preference and selected variables for participants who completed the survey (N = 561; RR>1 indicates the group is more likely to choose paper SAQ).

	Unadjusted		Adjusted	
	RR	95% Confidence Interval	RR	95% Confidence Interval
10-Year Increase in Age	1.07	(1.02, 1.13)	1.08	(1.03, 1.13)
Race				
Non-Hispanic White	1.00	Ref	1.00	Ref
Non-White*	1.29	(1.11, 1.50)	1.26	(1.08, 1.47)
Gender				
Female	1.00	Ref	–	–
Male	0.91	(0.79, 1.05)		
Language				
English	1.00	Ref	–	–
Spanish	1.19	(1.03, 1.37)		
Other	0.99	(0.73, 1.36)		
Education				
Some College or More	1.00	Ref	1.00	Ref
HS Degree or Less	1.35	(1.19, 1.53)	1.25	(1.09, 1.43)
Employment				
Full-Time	1.00	Ref	–	–
Part-Time	1.11	(0.90, 1.37)		
Student	1.32	(1.02, 1.69)		
Retired	0.82	(0.58, 1.14)		
Unemployed	1.27	(1.08, 1.50)		
Income				
$20K+	1.00	Ref	–	–
<$20K	1.12	(0.97, 1.30)		
Insurance				
Private	1.00	Ref	–	–
Medicare	1.06	(0.87, 1.27)		
Medicaid	1.26	(1.06, 1.49)		
Uninsured	0.88	(0.65, 1.18)		
Other	0.91	(0.74, 1.12)		
Number of Chronic Diseases				
0	1.00	Ref	–	–
1	1.22	(1.05, 1.41)		
2+	1.00	(0.82, 1.22)		
HIV Knowledge – 10% Increase in Number Correct	0.96	(0.93, 0.99)	–	–
Depression				
No	1.00	Ref	–	–
Yes	1.06	0.91, 1.25		
Alcohol Dependence via AUDIT				
No	1.00	Ref	–	–
Yes	1.06	0.87, 1.30		
Drug Use				
No	1.00	Ref	–	–
Yes	1.04	0.89, 1.22		

* Non-White race includes all Non-Hispanic Blacks, Hispanics, and Non-Hispanic others.

### Impact of Survey Modality on Unusable Data

**Figure 1:**
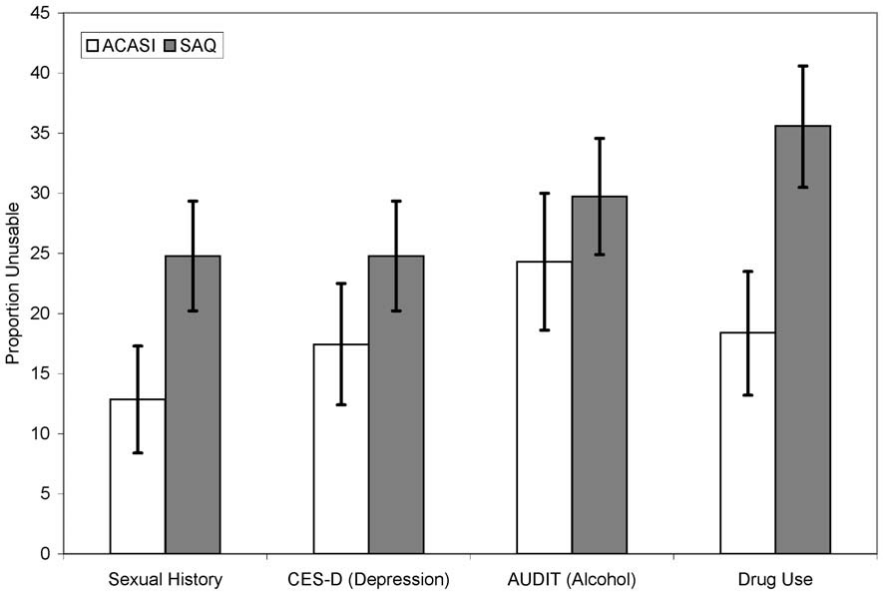
Proportion of unusable data from the survey by threshold for unusable data and section of the questionnaire. Unusable data was defined as having responded to less than 75% of the questions for the selected domains of the survey. The dark gray bars represent participants who chose SAQ, while the white bars represent participants who chose the ACASI version of the survey. Error bars in Figure 1 represent the 95% confidence interval. 95% confidence intervals that do not overlap imply a statistical difference in unusable data by survey modality for that particular domain. CES-D: Center for Epidemiologic Studies Depression Scale. AUDIT: Alcohol Use Disorders Identification. STD: Sexually Transmitted Disease. ACASI: Audio Computer Assisted Self-Interview. SAQ: Self-Administered Questionnaire.

Results examining the impact of survey modality on the magnitude of unusable data were examined across four different domains (sexual history; depression; alcohol use; and illicit drug use) are displayed in Figure 1 and Table 3. Figure 1 displays the proportion of unusable data by section of the survey for our base case threshold of unusable data of 75%. For the sexual history and depression domains the proportion of unusable data were 25% and 25% for the SAQ modality compared to 13% and 17% for the ACASI modality respectively. The smallest difference between the two modalities occurred in the alcohol use domain. Twenty-four percent of the participants who chose ACASI had unusable data for this domain compared to 30% for those who chose SAQ. The greatest disparity between the two modalities with respect to unusable data was for the illicit drug use domain with 18% of ACASI participants having unusable data compared to 36% of the SAQ participants.

**Table 3:** Results of the multivariate analysis examining the association of questionnaire domain and unusable data.

Domain	Unadjusted	Adjusted for Quintile of Propensity Score
Sexual Behavior	1.93 (1.30, 2.86)	1.57 (0.99, 2.48)
CES-D (Depression)	1.42 (1.01, 2.00)	1.51 (1.01, 2.25)
AUDIT (Alcohol)	1.22 (0.92, 1.63)	1.27 (0.90, 1.78)
Drug Use	1.94 (1.42, 2.65)	2.04 (1.43, 2.90)

CES-D: Center for Epidemiologic Studies Depression Scale.

AUDIT: Alcohol Use Disorders Identification.

STDs: Sexually Transmitted Diseases.

ACASI: Audio Computer Assisted Self-Interview.

SAQ: Self-Administered Questionnaire.

Relative risks >1 indicate that SAQ users were more likely to have unusable data than ACASI users.

Unusable data is defined as answering less than 75% of the questions for the particular domain.

Table 3 displays relative risks having unusable data for the four domains at the base case threshold of 75%. The results of an unadjusted analysis and an analysis that adjusts for the quintile of the propensity score are displayed. In all cases the estimated RR was greater than 1, indicating that participants who chose the SAQ version of the survey were more likely to have unusable data in each of the four domains than those who chose the ACASI version of the survey. In the unadjusted analysis this finding was statistically significant in the sexual behavior, depression, and illicit drug use domains. The finding was not statistically significant for the alcohol use domain. In the analysis that adjusted for the quintile of the propensity score the finding was statistically significant in the depression and illicit drug use domains but not in the sexual behavior or alcohol use domain (Table 3).

Results were similar for our sensitivity analysis using 90% as the threshold for unusable data. The proportion of unusable data for the ACASI version of the survey was 13%, 18%, 26%, and 19% for the sexual behavior, depression, alcohol use, and illicit drug use domains, respectively. This proportion was higher for the SAQ version of the survey with 28%, 28%, 31%, and 38% unusable data across the four domains, respectively. When we adjusted for the quintile of the propensity score, survey modality significantly impacted data quality in the sexual behavior, depression, and illicit drug use domains, but not the alcohol use domain.

## Discussion

ACASI is a popular mode of data collection in clinical research settings, especially when the survey includes sensitive questions. In the context of a randomized trial, we initially only offered the ACASI version of our survey, but anecdotal experience suggested that participants may prefer to complete the survey via SAQ. Thus, participants were provided the option of completing our survey via ACASI or the more traditional pen and paper SAQ. We provided this option so that we could more formally address the question of participant preference of survey type and the data quality arising from each modality. In the context of randomized trial of universal screening for HIV in the emergency department, we found that patient age, race, and education were associated with patient preference for survey modality. For every 10-year increase in age, patients were 8% more likely to favor the SAQ version over ACASI. Non-white participants were 26% more likely to choose SAQ over ACASI and those with a high school degree or less were 25% more likely to choose the SAQ over ACASI. Because surveys were offered prior to testing, HIV rapid test results should have little influence on survey preference.

Despite the benefit of increased preference for the SAQ version of the survey, using this modality had its tradeoffs. Adjusted relative risks of having more unusable data with the SAQ, as opposed to ACASI, ranged from 1.3 (95% CI: 0.9, 1.8) (alcohol consumption domain) to 2.0 (95% CI: 1.4, 2.9) (illicit drug use domain), indicating that the SAQ version of the survey yielded more unusable data. We found that the SAQ version yielded more unusable data than the ACASI version across all four domains assessing sensitive information. This finding was robust regardless of the threshold that was used for defining unusable data or adjustments for the propensity score, a method of adjusting for multiple potential confounders [24]. The implications of these findings are substantial. The proportion of participants with unusable data ranged from 25 to 36% among those who chose the SAQ when the threshold for having unusable data was less than 75% complete. This relationship existed across critical domains ranging from sexual risk, alcohol and drug use to depression. Incomplete data makes it challenging, if not impossible, for researchers to use this information.

When offered the choice of answering our survey via SAQ or ACASI, we found that patients were more likely to choose the SAQ. These findings were especially true among less educated and older participants. However, this is the only study to our knowledge that examined patient choice of survey modality in the ED. It is possible that the laptop which delivered the ACASI proved to be too cumbersome for our participants and that the use of a handheld device may be more efficient and acceptable in this setting. However, we specifically chose the laptop to avoid the disappearance (theft) of devices, which we felt was reasonable given the busy ED setting. We also found that ACASI did provide the most complete data, as other studies have found [2], [6]–[9].

There are several limitations to our study. First, the primary objective of our analysis, while examined within the context of a RCT, was not assessed with a randomized design. It is possible that patients who chose the SAQ would have accepted the ACASI if no other alternative was given to them and vice versa. Also, we were unable to control for unmeasured confounders that might obscure the association between patient preference and education. Such measures for which we were not able to control include reading and computer literacy. While these measures are likely correlated with level of education, it may be important to understand how these two factors jointly affect the choice of survey modality. Another limitation of our study is that exit interviews were not conducted, which might have provided insights into barriers of ACASI administration in the ED. Lastly, two of the domains (sexual behavior and illicit drug use) in which we looked at the impact of survey modality on data quality were just a group of questions about specific topic and were not measured using validated scales. Despite these limitations, our study is unique in its setting, as no other study has compared ACASI and SAQ in the ED where an increasing number of patients are being tested for HIV [25]–[30].

We found that age, race, and education play an important role in survey preference. While more patients preferred the SAQ version of our survey over the ACASI, the ACASI version provided more complete data with regard to sections that cover sensitive topics. Further research should assess how strong the preference for SAQ over ACASI is using a randomized design. Also, research on novel approaches that maximize ED patients' comfort with ACASI would be of great benefit because the amount of missing data in the SAQ may limit its utility.

## Supporting Information

Appendix S1 This document is the paper version of the survey.
(0.21 MB DOC)
