# Supplementary material for: Does Modality of Survey Administration Impact Data Quality: Audio Computer Assisted Self Interview (ACASI) Versus Self-Administered Pen and Paper?
Source: PLoS One. 2010 Jan 15;5(1):e8728. doi: 10.1371/journal.pone.0008728 (PMC2806918; doi:10.1371/journal.pone.0008728)
Supplement: Appendix S1 — This document is the paper version of the survey. (0.21 MB DOC) [file pone.0008728.s001.doc]

**Patient ID #**_______________________

A. We will begin the questionnaire with a few questions about your thoughts regarding your need and desire for an HIV test.

A.1. I do not want an HIV test because I am not at risk and I do not need to be tested.

1 Strongly disagree

2 Disagree

3 Undecided

4 Agree

5 Strongly agree

A.2. It might be worth my getting an HIV test.

1 Strongly disagree

2 Disagree

3 Undecided

4 Agree

5 Strongly agree

A.3. Based on my risk for HIV infection, I think I should be HIV tested.

1 Strongly disagree

2 Disagree

3 Undecided

4 Agree

5 Strongly agree

**B. The questionnaire continues with some questions to assess your knowledge about HIV, the virus that causes AIDS.**

|  | **True** | **False** | **I don’t know** |
| --- | --- | --- | --- |
| B.1. Coughing and sneezing DO NOT spread HIV. | T | F | DK |
| B.2. A person can get HIV by sharing a glass of water with someone who has HIV. | T | F | DK |
| B.3. Pulling out the penis before a man climaxes/cums keeps his partner from getting HIV during sex. | T | F | DK |
| B.4. A woman can get HIV if she has anal sex with a man. | T | F | DK |
| B.5. Showering or washing one’s genitals/private parts, after sex keeps a person from getting HIV. | T | F | DK |
| B.6. All pregnant women with HIV will have babies born with AIDS. | T | F | DK |
| B.7. People who have been infected with HIV quickly show serious signs of being infected. | T | F | DK |
| B.8. There is a vaccine that can stop adults from getting HIV. | T | F | DK |
| B.9. People are likely to get HIV by deep kissing, putting their tongue in their partner’s mouth, if their partner has HIV. | T | F | DK |
| B.10. A woman cannot get HIV if she has sex during her period. | T | F | DK |
| B.11. There is a female condom that can help decrease a woman’s chance of getting HIV. | T | F | DK |
| B.12. A natural skin condom works better against HIV than does a latex condom. | T | F | DK |
| B.13. A person will NOT get HIV if she or he is taking antibiotics. | T | F | DK |
| B.14. Having sex with more than one partner can increase a person’s chance of being infected with HIV. | T | F | DK |
| B.15. Taking a test for HIV one week after having sex will tell a person if she or he has HIV. | T | F | DK |
| B.16. A person can get HIV by sitting in a hot tub or a swimming pool with a person who has HIV. | T | F | DK |
| B.17. A person can get HIV from oral sex. | T | F | DK |
| B.18. Using Vaseline or baby oil with condoms lowers the chance of getting HIV. | T | F | DK |

**C. Now the questionnaire continues with some basic questions about who you are and your state of health.**

C.1. Are you male or female?

1 Male _2_ Female

C.2. Which would you say BEST describes your religious background?

1 Christian

2 Jewish

3 Muslim

4 Buddhist

5 Hinduist

6 Other

C.3. What is your highest level of education?

1 Less than a high school degree

If you checked 1: Can you read? _1_ Yes _2_ No

2 High school degree/GED

3 Some college

4 College degree

5 Some post-college

6 Graduate/professional degree

C.4. How far did you travel to come to the BWH Emergency Department today:

1 Less than 1 mile

2 1-5 miles

3 5-10 miles

4 10-20 miles

5 More than 20 miles

C.5. Are you currently working?

1 Full-time

2 Part-time

3 Student

4 Retired

5 No, unemployed

C.6. What type of insurance do you have? (Please check all that apply.)

1 Uninsured

2 Medicare

3 Medicaid (also called MassHealth)

4 Private (for example, Blue Cross, Tufts, Harvard Pilgrim, GIC, Humana,

Kaiser Permanente etc.)

5 Other

C.7. What is your annual household income?

1 Less than $20,000

2 $20,000-$50,000

3 $50,000-$100,000

4 More than $100,000

C.8. Did someone help you decide to come and seek care in the Emergency Department today? (Please check all that apply.)

1 Yes, spouse

2 Yes, other family member

3 Yes, friend

4 Yes, primary care physician

5 No

C.9. If you had to return to this hospital tomorrow for a follow-up appointment, what would be the MOST LIKELY WAY you would get here?

0 I would walk

1 I would drive myself

2 Bus or T

3 Taxi

4 A friend or family member would take me

5 I would likely miss my appointment

C.10. Please check off all chronic diseases you have previously been diagnosed with.

a Diabetes (high sugar)

b Hypertension (high blood pressure)

c Heart Disease

d Stroke

e Asthma/Bronchitis/Emphysema

f Cancer

g Psychiatric Disorder

h Other

i None

C.11. When was the last time you saw a health care professional for any reason:

1 Less than 1 week ago

2 Less than 1 month ago

3 Less than 1 year ago

4 Within the last 5 years

5 More than 5 years ago

C.12. Do you have a primary care provider?
1 Yes _2_ No _3_ Don’t know

C.13. To the best of your knowledge, are all of your vaccinations up-to-date?

1 Yes _2_ No _3_ Don’t know

C.14. To the best of your knowledge, are all of your recommended screening procedures up-to-date (eg. colonoscopy, pap smear, mammogram)?

1 Yes _2_ No _3_ Don’t know

C.15. Please check off all chronic diseases that a family member (biological mother, father, or siblings) has been diagnosed with?

a Diabetes (high sugar)

b Hypertension (high blood pressure)

c Heart Disease

d Stroke

e Asthma/Bronchitis/Emphysema

f Cancer

g Psychiatric Disorder

h HIV

i Other

j None

We are now going to ask some questions about whether you have ever had an HIV test.

D.1. Have you ever been tested for HIV, the virus that causes AIDS?

1 Yes _2_ No

D.2. When were you last tested? (month/year) **___**______/_________

D.3. What were the results?

1 My last test was negative

2 My last test was positive

3 My last test was indeterminate

4 I was tested but I did not receive my results.

Now, the questionnaire continues with some more personal questions about how often and with whom you have sex. For these questions, we consider “sex” to mean penetration or exchange of bodily fluids either through oral, anal, or vaginal contact. We hope that you will answer these questions but if there are any you are uncomfortable with or do not understand, please feel free to skip them. Your results will, of course, be kept strictly confidential and no one will be able to link you to your answers.

E.1. How many different sexual partners (including oral, anal or vaginal) have you had in the past 12 months? _______________ *(If zero, skip to question E.5)*

E.2. How many different sexual partners (including oral, anal or vaginal) have you had in the past 3 months? ______________

E.3a. **THIS QUESTION IS FOR MEN ONLY**: In the last year, how would you describe your sexual practices?

1 I only have sex with men

2 I mostly have sex with men but occasionally have sex with women

3 I have sex equally with both men and women

4 I mostly have sex with women but occasionally have sex with men

5 I only have sex with women

E.3b. **THIS QUESTION IS FOR** **WOMEN ONLY**: In the past year, how would you describe your sexual practices?

1 I only have sex with women

2 I mostly have sex with women but I occasionally have sex with men

3 I have sex equally with both women and men

4 I mostly have sex with men but I occasionally have sex with women

5 I only have sex with men

E.4. I or my partner use a condom:

1 always

2 most of the time

3 sometimes

4 never

E.5. Have you ever had sex with a person who was HIV-positive or had AIDS?

1 Yes, I had sex with a person diagnosed with HIV or AIDS

2 No, not that I know of

E.6. Have you ever been incarcerated (in prison)?

1 Yes _2_ No

E.7. Have you ever had sex with someone who has been incarcerated (in prison)?

1 Yes

2 No

3 I don’t know

These next several questions ask about your mood.

**Below is a list of some of the ways you may have felt or behaved. Please indicate how often you have felt this way by checking the appropriate box for each question.**

***During the past week…***

F.1. I was bothered by things that usually don’t bother me

1 Rarely or none of the time (less than 1 day)

2 Some or a little of the time (1-2 days)

3 Occasionally or a moderate amount of time (3-4 days)

4 All of the time (5-7 days)

F.2. I did not feel like eating; my appetite was poor

1 Rarely or none of the time (less than 1 day)

2 Some or a little of the time (1-2 days)

3 Occasionally or a moderate amount of time (3-4 days)

4 All of the time (5-7 days)

F.3. I felt that I could not shake off the blues even with help from my family

1 Rarely or none of the time (less than 1 day)

2 Some or a little of the time (1-2 days)

3 Occasionally or a moderate amount of time (3-4 days)

4 All of the time (5-7 days)

F.4. I felt that I was just as good as other people

1 Rarely or none of the time (less than 1 day)

2 Some or a little of the time (1-2 days)

3 Occasionally or a moderate amount of time (3-4 days)

4 All of the time (5-7 days)

F.5. I had trouble keeping my mind on what I was doing

1 Rarely or none of the time (less than 1 day)

2 Some or a little of the time (1-2 days)

3 Occasionally or a moderate amount of time (3-4 days)

4 All of the time (5-7 days)

F.6. I felt depressed

1 Rarely or none of the time (less than 1 day)

2 Some or a little of the time (1-2 days)

3 Occasionally or a moderate amount of time (3-4 days)

4 All of the time (5-7 days)

F.7. I felt that everything I did was an effort

1 Rarely or none of the time (less than 1 day)

2 Some or a little of the time (1-2 days)

3 Occasionally or a moderate amount of time (3-4 days)

4 All of the time (5-7 days)

F.8. I felt hopeful about the future

1 Rarely or none of the time (less than 1 day)

2 Some or a little of the time (1-2 days)

3 Occasionally or a moderate amount of time (3-4 days)

4 All of the time (5-7 days)

F.9. I thought my life had been a failure

1 Rarely or none of the time (less than 1 day)

2 Some or a little of the time (1-2 days)

3 Occasionally or a moderate amount of time (3-4 days)

4 All of the time (5-7 days)

F.10. I felt fearful

1 Rarely or none of the time (less than 1 day)

2 Some or a little of the time (1-2 days)

3 Occasionally or a moderate amount of time (3-4 days)

4 All of the time (5-7 days)

F.11. My sleep was restless

1 Rarely or none of the time (less than 1 day)

2 Some or a little of the time (1-2 days)

3 Occasionally or a moderate amount of time (3-4 days)

4 All of the time (5-7 days)

F.12. I was happy

1 Rarely or none of the time (less than 1 day)

2 Some or a little of the time (1-2 days)

3 Occasionally or a moderate amount of time (3-4 days)

4 All of the time (5-7 days)

F.13. I talked less than usual

1 Rarely or none of the time (less than 1 day)

2 Some or a little of the time (1-2 days)

3 Occasionally or a moderate amount of time (3-4 days)

4 All of the time (5-7 days)

F.14. I felt lonely

1 Rarely or none of the time (less than 1 day)

2 Some or a little of the time (1-2 days)

3 Occasionally or a moderate amount of time (3-4 days)

4 All of the time (5-7 days)

F.15. People were unfriendly

1 Rarely or none of the time (less than 1 day)

2 Some or a little of the time (1-2 days)

3 Occasionally or a moderate amount of time (3-4 days)

4 All of the time (5-7 days)

F.16. I enjoyed life

1 Rarely or none of the time (less than 1 day)

2 Some or a little of the time (1-2 days)

3 Occasionally or a moderate amount of time (3-4 days)

4 All of the time (5-7 days)

F.17. I had crying spells

1 Rarely or none of the time (less than 1 day)

2 Some or a little of the time (1-2 days)

3 Occasionally or a moderate amount of time (3-4 days)

4 All of the time (5-7 days)

F.18. I felt sad

1 Rarely or none of the time (less than 1 day)

2 Some or a little of the time (1-2 days)

3 Occasionally or a moderate amount of time (3-4 days)

4 All of the time (5-7 days)

F.19. I felt that people disliked me

1 Rarely or none of the time (less than 1 day)

2 Some or a little of the time (1-2 days)

3 Occasionally or a moderate amount of time (3-4 days)

4 All of the time (5-7 days)

F.20. I could not “get going”

1 Rarely or none of the time (less than 1 day)

2 Some or a little of the time (1-2 days)

3 Occasionally or a moderate amount of time (3-4 days)

4 All of the time (5-7 days)

**For the following questions, consider this statement:**

**In your life, have you ever had any experience that was so frightening, horrible, or upsetting that, *in the past month*, you:**

F.21. Have had nightmares about it or thought about it when you did not want to?

1 Yes

2 No

F.22. Tried hard not to think about it or went out of your way to avoid situations that remind you of it?

1 Yes

2 No

F.23. Were constantly on guard, watchful, or easily startled?

1 Yes

2 No

F.24. Felt numb or detached from others, activities, or your surroundings?

1 Yes

2 No

We are nearing the end of the questionnaire. Now we are going to ask some very personal questions about any smoking, alcohol and drug use you may have. Again, we hope that you will answer the questions but please feel free to skip any you are uncomfortable with. Your results will be kept strictly confidential and will not be able to be linked to your personal identity.

G.1. Do you currently smoke cigarettes (at least one cigarette per day)?

2 No *[if “No”, go to question G.2]*

1 Yes *[if “Yes”, go to question G.1a]*

G.1a. How many cigarettes per day (on average) do you smoke? ______

*[go to question G.3]*

G.2. Have you ever smoked at least one cigarette per day?

2 No *[if “No”, go to question G.3]*

1 Yes *[if “Yes”, go to question G.2a]*

G.2a. How many cigarettes per day (on average) did you smoke? ­­_______

G.2b. When did you stop? ______

The next ten items refer to your drinking habits. For each item, indicate the statement that is most true for you by checking the box next to the item.

Drink Definitions: For the purpose of this screening test, one unit of alcohol is defined as follows: 1) a single small glass of beer (8 ounces; ½ pint), 2) a single shot of liquor/spirits, 3) a single glass of wine.

*During the past year…*

G.3. How often do you have a drink containing alcohol?

1 Never

2 Monthly or less

3 2-4 times a month

4 2-3 times a week

5 4 or more times a week

G.4. How many units of alcohol do you drink on a typical day when you are drinking? (see “Drink Definitions” above)

1 1 or 2

2 3 or 4

3 5 or 6

4 7, 8 or 9

5 10 or more

G.5. How often do you have six or more drinks of alcohol on one occasion?

1 Never

2 Less than monthly

3 Monthly

4 Weekly

5 Daily or almost daily

G.6. How often during the last year have you found that you were not able to stop drinking once you had started?

1 Never

2 Less than monthly

3 Monthly

4 Weekly

5 Daily or almost daily

G.7. How often during the last year have you failed to do what was normally expected from you because of drinking?

1 Never

2 Less than monthly

3 Monthly

4 Weekly

5 Daily or almost daily

G.8. How often during the last year have you needed a first drink in the morning to get yourself going after a heavy drinking session?

1 Never

2 Less than monthly

3 Monthly

4 Weekly

5 Daily or almost daily

G.9. How often during the last year have you had a feeling of guilt or remorse after drinking?

1 Never

2 Less than monthly

3 Monthly

4 Weekly

5 Daily or almost daily

G.10. How often during the last year have you been unable to remember what happened the night before because you had been drinking?

1 Never

2 Less than monthly

3 Monthly

4 Weekly

5 Daily or almost daily

G.11. Have you or someone else been injured as a result of your drinking?

0 No

1 Yes, but not in the last year

2 Yes, during the last year

G.12. Has a relative or friend or doctor or another health worker been concerned about your drinking or suggested you cut down?

0 No

1 Yes, but not in the last year

2 Yes, during the last year

G.13. Have you used/tried any of the following drugs? (Please check all that apply and then click “Next Question”.)

|  | Never | Once | Occasionally | Monthly | More than once per month |
| --- | --- | --- | --- | --- | --- |
| 1. Heroin | 1 | 2 | 3 | 4 | 5 |
| 1. Cocaine, Crack | 1 | 2 | 3 | 4 | 5 |
| 1. Speed | 1 | 2 | 3 | 4 | 5 |
| 1. Oxycontin/other narcotics | 1 | 2 | 3 | 4 | 5 |
| 1. Poppers | 1 | 2 | 3 | 4 | 5 |
| 1. Pot | 1 | 2 | 3 | 4 | 5 |
| 1. Crystal Methamphetamine | 1 | 2 | 3 | 4 | 5 |
| 1. LSD | 1 | 2 | 3 | 4 | 5 |
| 1. Ecstasy | 1 | 2 | 3 | 4 | 5 |
| 1. Other | 1 | 2 | 3 | 4 | 5 |

G.14. If you have used injectable non-prescription drugs (recreational drugs like heroin, cocaine, speed, etc) or used injectable steroids, how often do you share needles or works?

1 I have used these drugs, but I have never shared needles or works

2 I have used these drugs and shared needles or works, and I rinsed the needles or works with bleach every time

3 I have used these drugs and shared needles or works, and I DID NOT rinse the needles or works with bleach every time

4 I have used these drugs and shared needles or works but I NEVER shoot behind someone else.

5 I never use these drugs.

G.15. Have you ever had sex with someone who uses or used recreational drugs?

1 Yes _2_ No, not that I know of

G.16. Have you ever been diagnosed with any of the following? Please check off as many as apply.

a Herpes

b Genital Warts (HPV)

c hepatitis B or C

d Chlamydia

e Gonorrhea

f Syphilis

g “Crabs”

h Other sexually transmitted disease(s)

i None

Thank you very much for your time in completing this survey and for your participation in the study. We assure you that your answers will be kept strictly confidential and will not be linked to your personal identity.

**To be filled out by the research assistant:** Did you assist the patient with filling out the questionnaire?

1 Yes _2_ No

**To be filled out by the research assistant:** Was the questionnaire filled out on paper or on the computer?

1 Computer _2_ Paper
